# Assessing the Cervicovaginal Microbiota in the Context of hrHPV Infections: Temporal Dynamics and Therapeutic Strategies

**DOI:** 10.1128/mbio.01619-22

**Published:** 2022-08-18

**Authors:** Mariano A. Molina, Britt A. Coenen, William P. J. Leenders, Karolina M. Andralojc, Martijn A. Huynen, Willem J. G. Melchers

**Affiliations:** a Department of Medical Microbiology, Radboud University Medical Center, Nijmegen, The Netherlands; b Department of Medical Microbiology, Radboud Institute for Molecular Life Sciences, Nijmegen, The Netherlands; c Department of Biochemistry, Radboud Institute for Molecular Life Sciences, Nijmegen, The Netherlands; d Center for Molecular and Biomolecular Informatics, Radboud Institute for Molecular Life Sciences, Nijmegen, The Netherlands; Tufts University School of Medicine; Icahn School of Medicine at Mount Sinai

**Keywords:** microbiome, cervicovaginal microbiota, microbial communities, CVM, CSTs, human papillomavirus, hrHPV

## Abstract

Cervical cancer is the third leading cause of female cancers globally, resulting in more than 300,000 deaths every year. The majority of all cervical cancers are caused by persistent infections with high-risk human papillomaviruses (hrHPV) that can progress to cancer via a series of premalignant lesions. Most women, however, clear this infection within a year, concomitant with disease regression. Both hrHPV clearance and disease regression have been associated with the composition of the cervicovaginal microenvironment, which is defined by the host immune system and the cervicovaginal microbiome (CVM). A healthy microbiome is generally characterized by a high abundance of *Lactobacillus* species, and a change in the composition may cause bacterial vaginosis (BV), which is associated with an increased susceptibility to persistent hrHPV infections and disease. In this review, the composition of the CVM is discussed, with emphasis on the possible causes that drive changes in the cervicovaginal microbiota in relation to hrHPV infections, disease progression, and disease regression. The literature search focused on the composition of the CVM and its correlation with hrHPV infections and neoplastic lesions as well as the current efforts to adjust the microbiome against adverse viral outcomes.

## INTRODUCTION

Cervical cancer is the third leading cause of female cancers globally, with approximately 570,000 cases and 300,000 deaths annually (1). High-risk human papillomaviruses (hrHPV) are the main cause of cervical intraepithelial neoplasia (CIN) and cervical carcinomas (2). hrHPVs are sexually transmitted, nonenveloped, double-stranded DNA viruses from the family *Papillomaviridae* that infect the basal cells of a variety of epithelial tissues, including the transition zone between the cervix and the endometrium (3). Under conditions of persistence, hrHPV can cause premalignant CIN, which can progress to invasive cancer, a process that can take 5 to 20 years (3). Cervical cancer screening programs aim to detect and remove these CIN lesions in a timely manner. Cervical cancer is highly prevalent in low-income and middle-income countries due to the lack of screening programs and the slow introduction of HPV vaccines (4). Additional risk factors to the disease include smoking, unhealthy lifestyle, and number of sexual partners (5, 6). Around 80% of all sexually active women will experience an HPV infection during their lives (7, 8). In the majority of the cases, the infection remains unnoticed, as the immune system is able to clear it (9–11). However, in some women, hrHPV will persist. A persistent infection with hrHPV is considered the most significant risk factor in the development of cervical carcinomas (1, 12), with integrated hrHPV DNA being present in almost all cervical cancer biopsy specimens (13, 14).

The cervicovaginal mucosa is the intrinsic defense against pathogens entering the vagina. It consists of a stratified squamous epithelium covered by a mucosal layer, which is constantly lubricated by cervicovaginal fluid. The cervicovaginal fluid is composed of many different elements secreted by cells and bacteria that are present in the vagina, and it contains products that are important for the protection of the vagina and the cervix, such as mucins and antimicrobial molecules, including B-defensins, lipocalin, elafin, secretory leukocyte protease inhibitors (SLPI), and immunoglobulins IgA and IgG (15, 16). Moreover, the cervicovaginal fluid helps to maintain the cervicovaginal microbiome (CVM). The CVM is structured into microbial communities that exist in a symbiotic relationship with the host and are often dominated by *Lactobacillus* species (17, 18). *Lactobacillus* species secrete lactic acid, maintain a low pH in the vagina, and adhere to epithelial surfaces, thereby preventing the adhesion of pathogenic bacteria to epithelial cells (19). The cervicovaginal ecosystem is also comprised of innate and adaptive immune cells, such as macrophages, neutrophils, NK cells, dendritic cells, Langerhans cells, T cells, and lymphocytes that regulate the immune response in the vagina (20).

The CVM has been overwhelmingly recognized as a candidate biomarker for the progression and regression of hrHPV infections (21). A healthy *Lactobacillus*-dominated microbiome prevents bacterial vaginosis (BV) and urogenital infections caused by yeasts, fungi, and human immunodeficiency virus (HIV) (22, 23). A highly diverse microbiota is associated with an increased risk of genital tract health problems, including hrHPV and HIV infections (24, 25). In this review, we discuss the composition and function of the lower genital tract microbiome, the CVM, and the effects of changes in the cervicovaginal environment on hrHPV infection outcomes.

## HUMAN PAPILLOMAVIRUS INFECTION AND LIFE CYCLE

The human papillomavirus family is divided into five genera (Alpha, Beta, Gamma, Mu, and Nu) based on DNA sequence analysis. They differ in life cycle characteristics and disease association (26, 27). The Beta, Gamma, Mu, and Nu genera are associated with asymptomatic infections that cause cutaneous lesions, while the Alpha type causes asymptomatic infections that result in mucosal and cutaneous lesions. The mucosal Alpha viruses are further divided into high-risk (hrHPV) and low-risk (lrHPV) genotypes based on their association with cancer (27). The lrHPV genotypes are generally associated with warts and do not typically cause neoplasia. In contrast, the hrHPV genotypes are associated with carcinomas, of which genotypes 16, 18, 31, 33, 35, 39, 45, 51, 52, 56, 58, 59, 66, and 68 are defined by the World Health Organization (WHO) as cancer-causing types. Overall, hrHPV genotypes 16 and 18 are responsible for 70% of women’s cervical cancer worldwide and therefore are considered the most oncogenic genotypes (28, 29).

An hrHPV infection is limited to the basal keratinocytes of several stratified epithelial tissues, including the transition zone between the cervix and the uterus in women, where it infects the basement membrane through microwounds and attaches to cells via the viral capsid proteins L1 and L2 (26). After internalization in basal epithelial cells (30) and following uncoating, the viral genome is initially retained as an extrachromosomal circular episome. The expression of the early genes E1 and E2 ensures that the viral DNA is maintained as an episome and facilitates the correct segregation of genomes during cell division (26, 31). The infected cells will go through natural keratinocyte differentiation in the transformation zone while carrying the viral genome. In an uninfected epithelium, basal cells exit the cell cycle soon after migrating into the suprabasal cell layer, where they undergo a process of terminal differentiation. However, when the expression of hrHPV proteins E6 and E7 is induced, cell cycle regulation and differentiation are disrupted (32). The expression of hrHPV E6 and E7 is often induced after the integration of the viral genome into the host genome. Interestingly, integration often leads to the deletion of essential viral genes for the synthesis of new viral particles, as integration mostly occurs in one of the early genes and disrupts its reading frames (33). The loss of E2, a repressor of the viral E6 and E7 gene promoters, will therefore increase the expression of the E6 and E7 genes (33, 34). Via interference with p53 and RB signaling, respectively, the E6 and E7 oncoproteins disrupt cell cycle control and induce genomic instability as a first step to cancer initiation (35, 36).

In productive hrHPV infections, the virus evades local immune responses by minimizing antigen production and by expressing the oncoproteins E6 and E7 (37, 38). In the early phase of infection, hrHPVs express a low abundance of E5, E6, and E7 proteins that are promptly translocated to the cell nucleus, thereby minimizing viral antigen presentation to the host immune cells present in the epithelial layer (37). In the late phase of infection, hrHPV capsid proteins are expressed. These are secreted toward the outer layer of the epithelium, where relatively few antigen-presenting cells are present, allowing immune escape (39). Likewise, the high binding affinity of the oncoproteins E6 and E7 to immune cells blocks immune-related gene expression and immune signaling pathways in infected keratinocytes (40). The impairment of immune responses in infected cells affects their capacity to activate local immune cells, resulting in an overall immunosuppressive environment that allows for carcinogenesis.

## COMPOSITION OF THE CERVICOVAGINAL MICROBIOME

**FIG 1:**
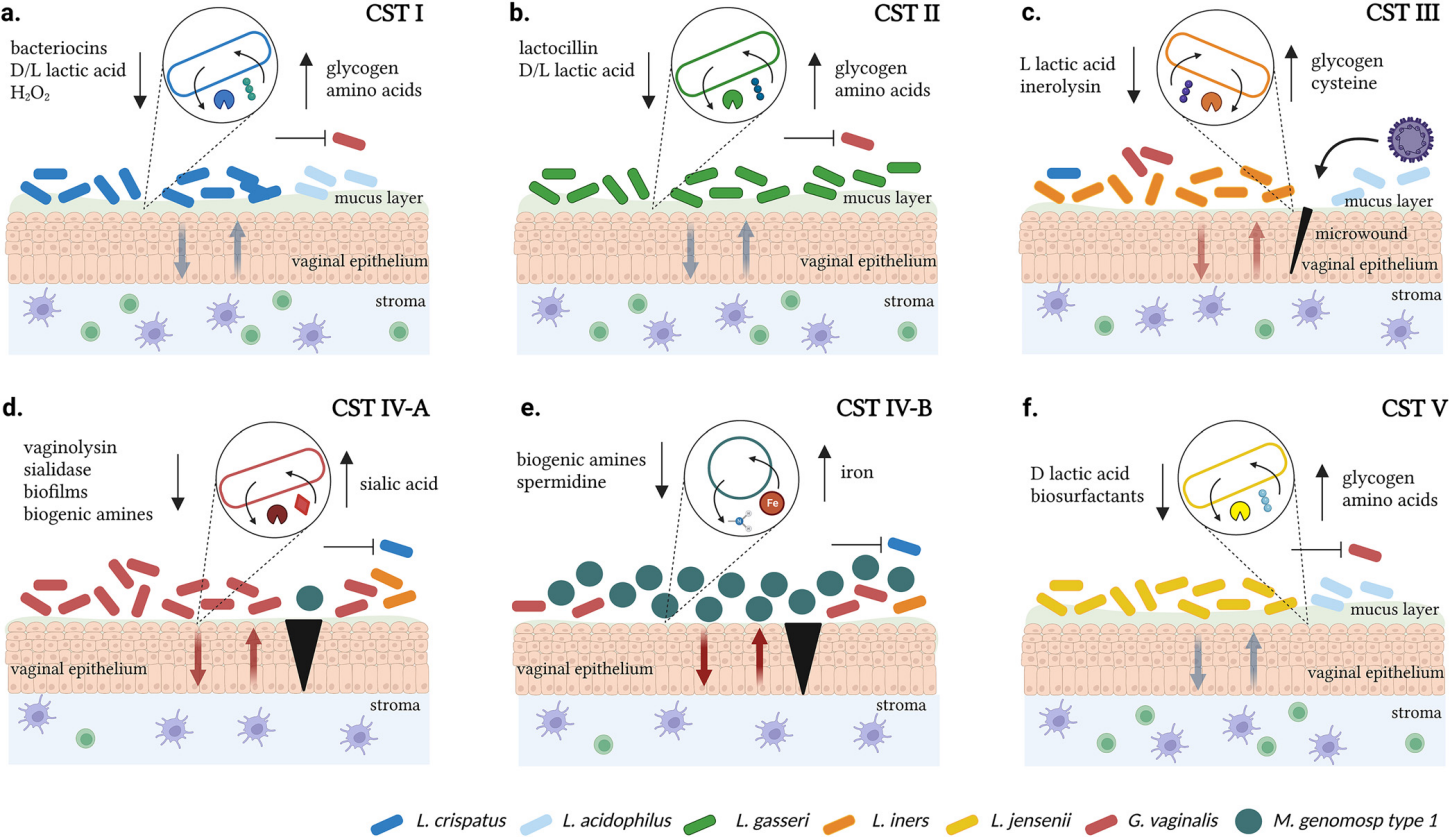
The biology of microbial communities in the cervicovaginal ecosystem. (A) Lactobacillus crispatus dominance (CST I, blue rods) in the cervicovaginal microbiota associates with a lower abundance for pathogenic bacteria (red rods). L. crispatus colonization leads to the production of l-and d-lactic acids, which acidifies the vaginal pH, and other products, such as H_2_O_2_ and bacteriocins that inhibit harmful bacteria. d-lactic acid and bacterial proteins modulate the host immune response toward an anti-inflammatory vaginal state. Glycogen, immune peptides, and amino acids found in the mucus layer serve as nutrient sources for the bacterium. (B) Lactobacillus gasseri dominance (CST II, green rods) in the cervicovaginal microbiota associates with a low abundance for pathogenic bacteria (red rods). L. gasseri produces l-and d-lactic acids, which acidify the vaginal pH, and bacteriocins, such as Lactocillin, that inhibit harmful bacteria. d-lactic acid and bacterial proteins modulate the host immune response toward an anti-inflammatory state. Glycogen and immune peptides found in the mucus layer serve as nutrient sources for the bacterium. (C) Lactobacillus iners dominance (CST III, orange rods) in the cervicovaginal microbiota associates with a high abundance of pathogenic bacteria and a low abundance of L. crispatus. *L. iners* produces only l-lactic acid, which does not properly regulate the vaginal pH, and inerolysin, which promotes bacterial adhesion in the mucus layer. l-lactic acid does not induce an anti-inflammatory vaginal state, and there is a higher susceptibility to a cytotoxic immune response in the vaginal epithelium that results in the disruption of the mucus layer. This imbalance may also lead to microwounds (marked in black) that pose a risk of viral infections. Glycogen and cysteine, an amino acid produced in the mucus layer, serve as nutrient sources for the bacterium. Gardnerella vaginalis dominance (D, red rods) and *Megasphaera genomosp type 1* dominance (E, green cocci), along with high diversity (CST IV), in the vaginal cervicovaginal microbiota associate with a high abundance for pathogenic anaerobes and a low abundance for *Lactobacillus*. *G. vaginalis* and *M. genomosp type 1* produce biogenic amines, which increase the vaginal pH. *G. vaginalis* also releases vaginolysin and sialidase, which lyse epithelia cells and break mucus sialoglycans, disturbing the mucus layer. Without an anti-inflammatory vaginal state, the bacteria induce a cytotoxic immune response that impairs the vaginal epithelium. This dysbiosis may also lead to microwounds that pose a risk of viral infections. Sialic acid and iron products serve as a nutrient source for *G. vaginalis* and *M. genomosp type 1*. (F) Lactobacillus jensenii dominance (CST V, yellow rods) in the cervicovaginal microbiota associates with a lower abundance of pathogenic bacteria (red rods). L. jensenii produces d-lactic acid, which acidifies the vaginal pH, and biosurfactants that inhibit the epithelial adhesion of harmful bacteria. d-lactic acid and bacterial proteins modulate the host immune response toward an anti-inflammatory vaginal state. Glycogen and amino acids found in the mucus layer serve as nutrient sources for the bacterium. An up arrow indicates uptake/consumption and a down arrow indicates release/production.

The CVM is typically composed of *Lactobacillus* species, which are Gram-positive, rod-shaped bacteria. *Lactobacillus* contribute to women's cervical health by maintaining a low pH (≤4.5) in the vagina through lactic acid production. A stable CVM inhibits colonization by harmful bacterial taxa through the production of biosurfactants, which prevent the attachment of such species to the vaginal epithelium, as well as bacteriocins, which eradicate closely related bacterial species (Fig. 1) (41). Furthermore, the CVM is characterized by microbial communities, which are groups of bacteria that establish symbiotic relationships in the CVM (42–45). These community state types (CSTs) are classified into five major groups: I, II, III, IV, and V, with CSTs I, II, III, and V presenting low diversity and having microbial dominance for Lactobacillus crispatus, Lactobacillus gasseri, Lactobacillus iners, and Lactobacillus jensenii, respectively. Colonization by Lactobacillus acidophilus further subdivides CSTs I and III into A and B subgroups (46, 47). CST IV is characterized by high diversity, *Lactobacillus* depletion, and a high abundance of BV-associated bacteria, such as Gardnerella vaginalis, *Megasphaera genomosp type 1*, Prevotella timonensis, and *Sneathia amnii* (17, 46, 48, 49).

## CST I

CST I microbiomes are stable cervicovaginal environments. L. crispatus dominance maintains a protective environment and mucus layer by producing bacteriocins and peroxidase that kill pathogenic bacteria. The bacterium also utilizes sugars, such as glycogen, to produce d- and l-lactic acids that stimulate beneficial immune responses in the mucus layer, lower the vaginal pH, and prevent the outgrow of anaerobic pathogens, such as *G. vaginalis* (Fig. 1A) (50–52). L. crispatus promotes an anti-inflammatory state that is characterized by the production of interleukins and proinflammatory cytokines as well as T cell activation (50, 53). The bacterium also uses the immune peptides elafin and S100A7, derived from the mucus layer, as an amino acid source (54). Since colonization by pathogenic species is inhibited in this ecosystem, the crosstalk between the vaginal epithelium and the stromal immune responses creates a balance suitable for *Lactobacillus* species and the mucus layer (Fig. 1A).

## CST II

CST II microbiomes are rarely observed in women and are relatively stable. L. gasseri adheres to the mucus layer through mucus-binding proteins and produces both d- and l-lactic acids by glycogen consumption, thereby maintaining acidic conditions in the cervicovaginal microenvironment (Fig. 1B) (52, 55, 56). L. gasseri also produces the bacteriocins gassericin A and lactocillin, the latter being active against Enterococcus faecalis and *G. vaginalis* and thus having a protective role in the vagina. Lactocillin is inactive against *Lactobacillus* species, which suggests a resistance against a compound that they commonly encounter in the CVM (57–59). L. gasseri also promotes an anti-inflammatory state in the mucus layer by modulating tumor necrosis factor-α and interleukin-1β expression and increases vaginal epithelial cell exfoliation (Fig. 1B) (60). CST II has the highest vaginal pH (between 4.5 and 5.0) among the *Lactobacillus*-dominated CSTs and consequently has been associated with a higher susceptibility to dysbiosis (61, 62).

## CST III

CST III microbiomes have a more unstable microenvironment than do CSTs I and II. *L. iners* is the dominant species in this CST, and it only produces l-lactic acid, which may lead to less control of the vaginal pH (52, 63) than is displayed by species that produces both d- and l-lactic acids and to a lesser protection against the colonization of strict anaerobes and other pathogens (Fig. 1C). *L. iners* is more capable of surviving and adapting to an environment with a wide pH range and to environmental conditions related to metabolic stress due to its expression of specific stress-associated genes that have not been found in other *Lactobacillus* species (64, 65). The production of inerolysin by *L. iners*, an enzyme responsible for adhesion, can result in the impairment of the mucus layer and epithelial cells, which can cause microwounds that are susceptible to viral infections (66). *L. iners* uses the amino acid cysteine, which is produced in the mucus layer, in its metabolic activities (67). The disturbance of the cervicovaginal ecosystem by CST III microbiomes can promote immune responses that cause cytotoxic effects in the epithelium and can result in a lower number of antigen-presenting cells (Fig. 1C) (66).

## CSTs IV-A AND IV-B

CST IV microbiomes are characterized by a high vaginal pH (≥5.0) and a high production of biogenic amines, such as putrescine, cadaverine, tyramine, agmatine, spermine, and trimethylamine that allow pathogenic bacteria to grow, thereby promoting dysbiosis and the inhibition of *Lactobacillus* species (68, 69). In CST IV-A microbiomes, where *G. vaginalis* is generally dominant, the bacterium releases vaginolysin and sialidase enzymes that may cause the lysis of epithelial cells and the hydrolysis of sialic acid residue from mucus sialoglycans in the cervicovaginal mucus, respectively, leading to the disruption of the mucus barrier membrane (Fig. 1D) (70, 71). *G. vaginalis* can also form biofilms that can shelter BV-associated bacteria from adverse conditions, thereby shaping the host immune responses (72). These conditions favor the infiltration of CD4^+^ T cells and the production of cytokines that are cytotoxic to epithelial cells and consequently promote the outgrowth of pathogenic anaerobes (Fig. 1D). Similarly, in CST IV-B, where *M. genomosp type 1* is dominant, the vaginal pH is also basic, and there is production of biogenic amines, including spermidines (Fig. 1E) (46, 73, 74). *Megasphaera* species also take up iron available in the mucus layer for their metabolic activities (74). Although less investigated so far, *M. genomosp type 1* is believed to downregulate the colonization of *Lactobacillus* species and promote a strong immune response, thereby disrupting the mucus layer and enhancing colonization by pathogenic anaerobes (Fig. 1E).

## CST V

CST V microbiomes are characterized by the dominance of L. jensenii. The bacterium produces d-lactic acid with glycogen as a nitrogen source, ensuring a protective acidic cervicovaginal environment (Fig. 1F) (52, 55). L. jensenii also makes biosurfactants that exhibit antimicrobial activity against bacterial pathogens (75–77). Similar to L. crispatus, L. jensenii promotes an anti-inflammatory state and uses the immune peptides elafin and S100A7, derived from the mucus layer, as an amino acid source (Fig. 1F) (54, 78).

## DYNAMICS OF COMMUNITY STATE TYPES

The CVM is a dynamic ecosystem, with CSTs transitioning between each other regularly, dependent on women’s cervicovaginal conditions. The most common transition observed is from CST III to CST IV (79). In general, many factors can influence the composition and changes within the CVM. For instance, the menstrual cycle, hygiene, vaginal douching, and hormonal changes all affect the pH balance of the vagina, possibly resulting in BV and CST IV. The prevalence of BV is high among reproductive-aged women globally, ranging from 23% to 29% (80, 81). Other studies have investigated the composition of the CVM and have identified even more CSTs than the classical five groups (46, 82, 83). For instance, the vaginal colonization by L. acidophilus is characteristic of the microbial subgroup B in both CST I and CST III (46, 61). The occurrence of L. acidophilus may promote a decrease in abundance for L. crispatus and *L. iners* in CSTs I and III, respectively, leading to a transitional state between both communities (46). It must be noted that microbiome clustering analysis is routinely performed with data from different techniques (16S rRNA gene sequencing, metagenomics) making data analyses nonuniform and complicating direct comparisons between different studies. Additionally, microbiome profiles may differ between different populations. Cheng et al. studied the composition of the CVM in young Swedish women and found that the majority of the subjects exhibited a CVM dominated by L. crispatus and *L. iners* or were *Lactobacillus*-depleted (32.7%, 30.4%, and 33.5%, respectively) (82). Likewise, Fettweis et al. studied the differences between the CVMs of African American women and Caucasian women (84). They found that in African American women, the most common profiles exhibited dominance for *L. iners*, followed by *G. vaginalis*, bacterial vaginosis-associated bacterium-1 (BVAB1), “other”, and L. crispatus. The most common profiles in Caucasian women were L. crispatus, followed by *L. iners* and *G. vaginalis* (84). In addition, Serrano et al. also showed that nonpregnant women of African ancestry had CVM profiles with *L. iners* as the most abundant bacterium, while nonpregnant Caucasian women had CSTs with L. crispatus as the most abundant bacterium (85).

## THE MICROBIOME IN hrHPV-POSITIVE WOMEN

**FIG 2:**
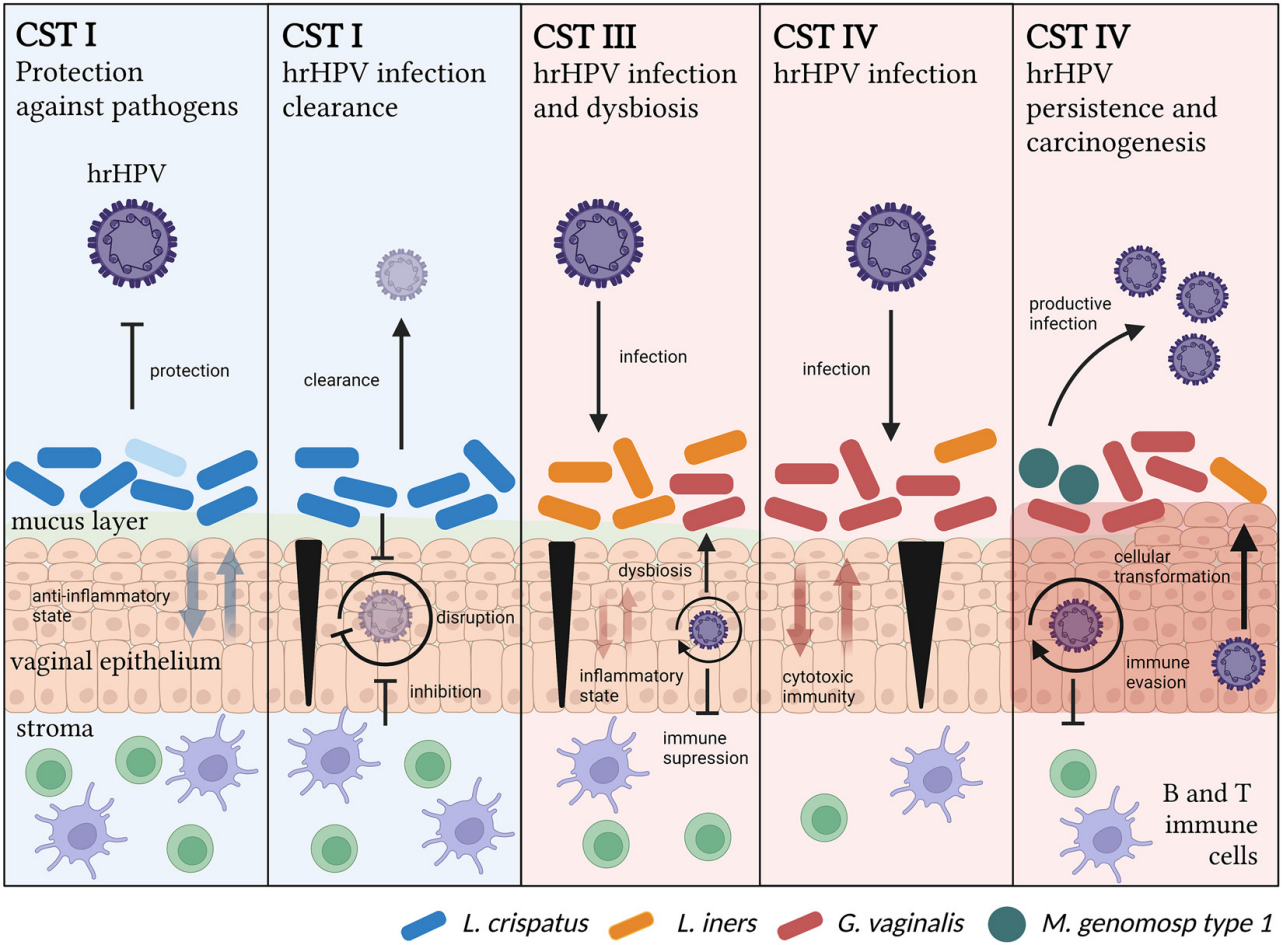
Temporal relationship between the microbiome and hrHPV. CST I is associated with protection against pathogens, including hrHPV, and hrHPV infection clearance. In this CST, the cervicovaginal microenvironment exhibits an anti-inflammatory state, allowing for effective immune responses against hrHPV and the disruption of its viral life cycle in the case of an infection. Alternatively, CST III is associated with susceptibility to hrHPV infections. In this community, the cervicovaginal microenvironment exhibits an increase in cytotoxic immune responses. Microwounds (marked in black) allow for productive hrHPV infections. The virus suppresses immune responses, which reduces the nutrient sources for *Lactobacillus* species and causes dysbiosis. CSTs IV are also associated with susceptibility to hrHPV infections, persistence, and carcinogenesis. In this CST, pathogenic bacteria induce the disruption of the mucus layer, which impairs anti-inflammatory immune responses and increases cytotoxic signals that destroy epithelial cells, thus allowing for productive viral infections. Dysbiosis, along with hrHPV persistence, eventually leads to cellular transformation and carcinogenesis.

Many studies have investigated the correlation between the CVM and hrHPV infection and have reported distinctive microbial signatures in all stages of cervical health and dysplasia. Lee et al. were the first to use 16S rRNA gene sequencing to investigate the impact of hrHPV infections on the CVM composition in a cross-sectional study, using the data of 912 women who participated in a Korean Twin Cohort study (86). They compared the CVMs of 23 hrHPV-positive premenopausal women, 4 of whom had developed CIN, and 27 hrHPV-negative premenopausal women, only one of whom had CIN. The average abundance of *Lactobacillus* species, such as L. crispatus, L. gasseri, *L. iners*, and L. jensenii, was much lower in the groups infected with hrHPV compared to the control group (Fig. 2). They also compared the CVMs of 16 premenopausal twin pairs, of which in 9 pairs, one was hrHPV-positive and one was hrHPV-negative. They found that in comparison to their uninfected twins, the hrHPV-positive women had a higher species diversity and a lower abundance for *Lactobacillus* species. The study identified *Sneathia* species as a microbiological marker of hrHPV infections, which are three times more frequent among hrHPV-positive women (Table 1).

**TABLE 1:** Summary of the microbiome association with hrHPV infections

Cervical conditions	Microbiome characteristics	References
Healthy cervix	CSTs I, II, III and V; L. crispatus, L. acidophilus	(47, 83, 104)
hrHPV infection	CSTs III, IV, *Sneathia* species	(86, 93, 94, 140–142)
hrHPV persistence	CSTs IV, IV-B	(92, 96)
hrHPV progression	CST IV	(91, 93, 94)
CIN1+	CSTs III, IV	(46, 47, 83, 94, 140)
Cervical cancer	CST IV, *Sneathia* and *Fusobacterium* species	(83, 94)
hrHPV clearance	CST II	(92)
CIN regression	CSTs I, II, III, and V	(96)

Aside from correlating with a higher incidence, prevalence, and persistence of hrHPV infections, BV has also been associated with the development of hrHPV-induced CIN (87–91). In a meta-analysis including 12 studies that involved 6,372 women in total, BV was found to be strongly associated with hrHPV infection (89). Similarly, Brotman et al. studied the vaginal microbiome and hrHPV presence in 32 reproductive-aged women, from whom they collected vaginal swabs twice weekly for 16 weeks in total (92). Using 16S rRNA gene sequencing, they clustered the CVM into six CSTs. They found that the largest proportion of hrHPV-positive samples was found with low abundance for *Lactobacillus* species (L. crispatus, L. jensenii, and L. gasseri), CST IV (71% hrHPV-positive), and CST III (72% hrHPV-positive). They further suggested that CST II was associated with the fastest remission rate of hrHPV infection, whereas CST IV-B was associated with the slowest remission rate (Table 1).

Since hrHPV persistence is necessary but insufficient for the formation of cervical neoplastic lesions, Mitra et al. investigated the structure of the CVM in correlation with CIN disease severity (93). They enrolled 169 women and classified them into 4 groups, depending on the severity of their lesions, and they compared the frequency of the different CSTs (I, II, III, IV, and V) to the CIN disease severity and healthy controls. They found that higher rates of CST IV (*Lactobacillus*-depleted) were associated with an increase in disease severity, with CST IV being observed twice as frequently in women with low-grade squamous intraepithelial lesions (LSIL), three times as frequently in women with high-grade squamous intraepithelial lesions (HSIL), and four times as frequently in women with invasive cervical cancer (ICC) compared to healthy controls. Furthermore, CST IV was more frequently observed in hrHPV-positive women than in hrHPV-negative women (Table 1) (93).

In a cross-sectional study by Audirac-Chalifour et al., the CVM and the cytokine profile were investigated at various cervical cancer stages (83). The CVM was determined via the high-throughput sequencing of 16S rRNA amplicons and classified in CSTs. In total, they classified eight different CSTs, according to bacterial dominance. Except for CST IV, all of the samples were clustered according to the histopathological diagnosis, and they found that CST I was mainly composed of hrHPV-negative women with noncervical lesions (Fig. 2). CST III was associated with hrHPV-positive women. *Sneathia*-dominated CSTs were predominantly found in women with squamous intraepithelial lesions (Table 1). A *Fusobacterium*-dominated CST was comprised of cervical cancer cases and showed higher levels of IL-4 and TGF-β1 mRNA compared to hrHPV-negative women (83).

Laniewski and his colleagues investigated the relationship between hrHPV, CVM composition, the level of genital immune mediators, and the severity of cervical lesions (94). Using data from Hispanic and non-Hispanic women with low-grade and high-grade cervical dysplasia, invasive cervical carcinoma, and healthy controls, they found that vaginal pH is associated with ethnicity and the severity of cervical lesions. This correlates with their finding that *Lactobacillus* dominance decreased with the severity of cervical lesions. Additionally, they found that hrHPV-positive women and women with LSIL, HSIL, or ICC showed an increase in bacterial taxa associated with BV. The study further reported that *Sneathia* was the only taxon significantly enriched in women with hrHPV infections as well as women with precancerous lesions and ICC. Interestingly, they also found *L. iners* to be enriched in hrHPV-positive women and women with LSIL or HSIL. Since *L. iners* can dominate the CVM of healthy women, and since studies showed that the *L. iners*-dominated CVM is more likely to transition to a more species-diverse CVM (Table 1) (95), *L. iners* may contribute to changes in the CVM composition that can eventually lead to disease progression. Similarly, Mitra et al. described the relationship between the CVM and the regression of untreated CIN2 lesions (96). They used vaginal samples from a cohort of 87 subjects with histologically confirmed, untreated CIN2 lesions to determine whether the CVM composition affects regression in a time frame of 2 years. They found that women with a *Lactobacillus*-dominant microbiome were more likely to have regressive disease after 1 year, whereas *Lactobacillus-*depleted microbiomes and the presence of pathogenic anerobic bacteria were associated with CIN2 persistence and slower regression. CST IV was also associated with a higher chance of hrHPV persistence after 1 or 2 years compared to women with CST I (Fig. 2).

There are several mechanisms that may explain why hrHPV is able to infect the basal keratinocytes and can lead to the development of cervical cancer in women with CST IV. Increased cell shedding and the reduced proliferation of the vaginal epithelium results in a thinner layer that the virus must get through in order to infect basal keratinocytes. During sexual intercourse, microwounds that facilitate the entry of hrHPV into the basal membrane can develop in the epithelial layer, while the mucus layer that protects the epithelium is reduced due to the depletion of *Lactobacillus* species (Fig. 2). Likewise, the anti-inflammatory environment that is normally created by lactic acid and the *Lactobacillus* species is absent in CST IV microbiomes, resulting in a dysregulated immune homeostasis in the vagina. The production of uncontrolled proinflammatory cytokines by vaginal epithelial cells and harmful bacteria results in inflammation, which requires constant cell renewal and, therefore, more cell proliferation, increasing the risk of hrHPV persistence and oncogenesis. This constant cell renewal and proliferation are then able to induce DNA replication stress, a mechanism which has been linked to hrHPV integration (97). The hrHPV then hijacks the host cell’s DNA damage response to replicate, a process which occurs adjacent to the regions in the host DNA that are susceptible to replication stress (14, 98, 99), which are also the preferential integration sites (100). However, this is not enough to facilitate integration into the host genome, as the integration must take place at a site in the DNA that supports viral oncogene expression and epigenetic modulation (101).

The mechanistic biology of CST shifts during hrHPV infections, and cervical disease remains poorly understood. However, Lebeau et al. recently proposed that hrHPV infection promotes a shift to CST IV by downregulating host immune responses, thereby resulting in a decrease of immune peptides, such as SLPI, S100A7, elafin, HβD2, and HβD4, in the mucosal layer (54, 102). Since these peptides are amino acid sources for the *Lactobacillus* species L. crispatus, L. gasseri, L. jensenii, and *L. iners* in the CVM and are used to sustain their growth and survival, a reduction of these amino acids will have a negative impact on the lactic bacteria, causing dysbiosis and a higher risk of hrHPV persistence. Likewise, Moscicki et al. observed that hrHPV-positive women who cleared the virus exhibited a higher abundance for *G. vaginalis* and, thus, CST IV, which they hypothesized occurred due to a switch from antimicrobial surveillance to an antiviral immune response (103, 104). This switch could result in a loss of microbial control, allowing for the expansion of pathogenic bacteria, such as *G. vaginalis* (103). This overall viral immunopathogenesis implies a timeline in which hrHPV infection precedes BV, which may occur when the virus is able to infect and persist in *Lactobacillus*-dominated CSTs, such as CST III. The CST III unstable microenvironment has a higher risk of the development of microwounds and detrimental immune responses, which could facilitate productive hrHPV infections and, thus, viral-induced dysbiosis (Fig. 2).

Metabolome analyses on the cervicovaginal environment have also revealed that hrHPV infection leads to a higher concentration of biogenic amines, sialidases (SNA), and phospholipids (105–107). In addition, hrHPV-positive women with CST III exhibit higher concentrations of biogenic amines and glycogen-related metabolites than do hrHPV-negative women with the same community (105). Likewise, hrHPV-positive women with CST IV microbiomes have lower concentrations of glutathione, glycogen, and phospholipid-related metabolites than do hrHPV-negative women with the same CST. In terms of HPV genotypes, hrHPV-infected women had lower concentrations of amino acids, lipids, and peptides compared with lrHPV-infected women (105). Moreover, compared to hrHPV-negative women, hrHPV-positive women with and without cervical dysplasia show a depletion of metabolites associated with taurine, glutamine, and lysine metabolisms, which correlates with the disruption of epithelial cellular growth and dysbiosis (108). Overall, metabolome studies show that there are key signatures on the cervicovaginal ecosystem that are associated with hrHPV infection and with the composition of the CVM, demonstrating that metabolic analyses of the cervicovaginal microenvironment may be a potential tool with which to discriminate hrHPV carcinogenesis and cervical cancer.

## MODULATING THE MICROBIOME AGAINST hrHPV INFECTIONS

**FIG 3:**
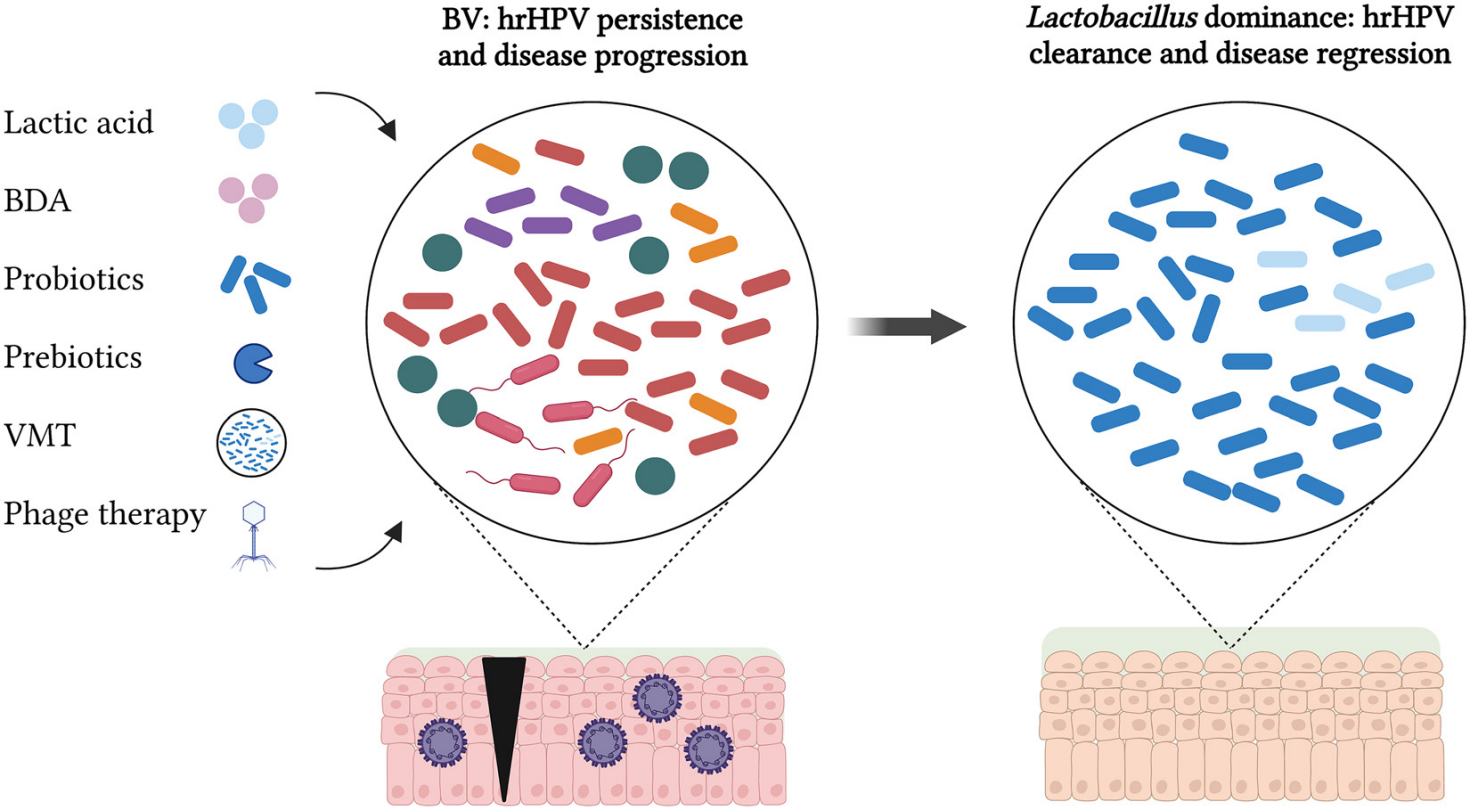
Therapeutic interventions in the microbiome for hrHPV infections. Current cervicovaginal microbiome-targeted therapies (CVMTs) include agents to lower the vaginal pH, such as probiotics and lactic acid. Potential CVMTs are the use of biofilm disruptive agents (BDA) and prebiotics to counteract harmful activities by BV-associated bacteria. Vaginal microbiome transplants (VMT) and phage therapy are promising therapies that can either replace or modify the vaginal microbiota, respectively. The successful application of these therapies in women with BV, hrHPV persistent infections, and cervical disease should result in a change of the microbial composition into a healthy, *Lactobacillus*-dominated microbiome, leading to hrHPV clearance and disease regression.

Attempts to establish long-lasting changes in the CVM have been mostly unsuccessful. Current treatments, such as antibiotics against BV (e.g., metronidazole) and estrogen therapy, have only a temporary effect on the CVM (109, 110). Although most of these approaches have been focused on achieving microbiome normalization with biofilm disruptive agents and lactic acid (111, 112), they have provided significant insights for the development of cervicovaginal microbiome-targeted therapies (CVMTs) during hrHPV infections (Fig. 3). Effective CVMTs should consider the protective effect of CST I and the detrimental effect of CST IV in the cervicovaginal environment. It is also important to consider the specific species that dominate the CVM, their metabolism sources, and the genetic material that they carry. This will facilitate the selection of suitable CVMTs that can be produced at large scale to treat hrHPV infections.

Among the widely studied CVMTs, we find the use of probiotics and prebiotics. Probiotics are live microorganisms that confer a health benefit when consumed in adequate amounts (113), while prebiotics are nutraceutical compounds that induce bacterial growth, the activity of probiotics, or beneficial endogenous microorganisms (Fig. 3). In a clinical trial, Ou et al. tested the use of Lactobacillus rhamnosus GR-1 and Lactobacillus reuteri RC-14 as oral treatments for hrHPV clearance (114). The probiotic was administered orally (one capsule daily) until hrHPV-negative test results were achieved. However, the study did not find an association of these two probiotics with hrHPV clearance, possibly due to the route of administration, the short-term applications, and the bacterial strains utilized (114). In contrast, a clinical trial by Palma et al. observed long-term restoration of the CVM ecosystem and hrHPV clearance in hrHPV-infected women following a vaginal treatment with Lactobacillus rhamnosus BMX 54 for up to 14 months (115). Lactobacillus crispatus CTV-05 (Lactin-V) has been successfully applied in BV treatment but not for hrHPV infections to date, representing a promising candidate for CVMTs against hrHPV (116). Similarly, there have been significant advances in the application of prebiotics as CVMTs. For instance, lactoferrin, an iron-binding glycoprotein, has been used as a possible treatment for BV and for preterm labor reduction (117–119). Lactoferrin has anti-inflammatory and antimicrobial activities as well as sequestrates iron, thus making it unavailable for the metabolism of harmful bacteria (120). In addition, cysteine inhibitors and the combination of the bacteriocins GasK7B α and β derived from a human intestinal strain of *Lactobacillus paragasseri* have been recently described as potential CVMTs that could prevent the growth of *L. iners* and the development of BV, and this could be applied against hrHPV infections, since both have been associated with hrHPV-induced cervical disease (67, 121).

Alternative potential CVMTs consist of either a partial modification or a complete replacement of the cervicovaginal microbiota through genetic therapy by CRISPR-Cas systems and by phages and vaginal microbiome transplants (VMT), respectively (Fig. 3). Microbiome transplants have been extensively studied in the context of the gut microbiome, and they have achieved effective treatment against recurrent Clostridioides difficile infections (122). VMT have encouraging results in animal models and in clinical trials for treating BV and thus could be applied as CVMTs for hrHPV infections (123, 124). Furthermore, Lam et al. reported the use of bacteriophages to deliver a programmable, exogenous CRISPR-Cas9 system to achieve the strain-specific depletion of fluorescently marked, isogenic E. coli strains in the gut microbiomes of mice (125). Phages have also been utilized to target *G. vaginalis* strains, reaching bacterial clearance *in vitro* and BV resolution *ex vivo* (126). Therefore, these genetic tools could be further studied in the genomic edition of the cervicovaginal microbiota during hrHPV infections to prevent viral persistence and carcinogenesis.

## INVESTIGATING THE MICROBIOME DURING hrHPV INFECTIONS

Assessing the role of the cervicovaginal microbiota in hrHPV infections and carcinogenesis has its limitations. However, novel approaches may assist in the study of the CVM. Microbiome alterations during cervical disease occur at the community and species levels, and even though amplicon-based techniques have facilitated a breakthrough of most of the CVM observations in the context of hrHPV infections, they generally cannot achieve high-resolution microbiome profiling. In contrast, next generation sequencing (NGS) approaches, such as shotgun metagenomics and circular-probe based RNA sequencing (ciRNAseq), can identify the most relevant microbes in the cervicovaginal microenvironment at the species level, thereby allowing for a better understanding of the CVM composition and hrHPV infections (47, 127). Likewise, in addition to cross-sectional studies, longitudinal studies are required to adequately evaluate the temporal dynamics of microbial communities and species. Microbial communities can naturally change over time, and particular species can induce microbial shifts that have been associated with hrHPV acquisition, viral persistence, and viral-induced dysbiosis. The longitudinal profiling of cervical smears from hrHPV-negative and hrHPV-positive women should elucidate such dynamics. These dynamics should be further validated through suitable *in vitro* and *in vivo* models that allow for accurate assessments into the biology of microbial species, CSTs, hosts, and hrHPV (54, 128). Available study models include organotypic raft cultures, 3D models of the human cervix, organoids, and the use of germfree animal models, such as zebrafish, mice, rats, and pigs (129, 130). Integration of the transcriptome, metabolome, and immunobiome into these studies should offer a deeper understanding of the CVM during hrHPV infections and cervical disease (131–133).

Exploring the interplay of the CVM in hrHPV infections also means considering interactions with other microbiomes. Although we focus this review on the lower genital tract microbiome, recent reports suggest the existence of an upper genital microbiome, the endometrial microbiome (EDM), which associates with endometrial diseases, such as endometriosis and cancer, and poor outcomes in assisted reproduction (134–136). Nevertheless, research on the EDM is still ongoing, and there is not clear evidence of established microbial communities or whether there is a relationship with the CVM and hrHPV infections (137). Similarly, the composition of the male genital microbiome has been associated with the protection and risk of sexually transmitted infections (STI) (138, 139), including hrHPV infections. Thus, additional analyses on the dynamics of the male and female genital microbiomes, as well as between the EDM and CVM during hrHPV acquisition and infections, are required to fully understand the role of the CVM in cervical health and disease (138).

## CONCLUSION

The composition and dynamics of the CVM have been associated with hrHPV infections and cervical disease behavior. In general, a beneficial cervicovaginal microbiome is characterized by *Lactobacillus* species, such as Lactobacillus crispatus (CST I), which protects the underlying epithelium and promotes hrHPV clearance and disease regression. In contrast, a disadvantageous microbiome is characterized by *Lactobacillus* depletion and high microbial diversity (CST IV), which support hrHPV infection, viral persistence, and carcinogenesis and correlates with disease progression and cervical cancer. The anti-inflammatory state promoted by the *Lactobacillus* species and the inhibition of bacterial species through the regulation of vaginal pH and bacteriocins are key features of CSTs that need to be considered when investigating the relationship between the CVM and hrHPV. High-throughput sequencing methods, as well as longitudinal and omics studies, including *in vitro* and *in vivo* experiments, will be essential for this purpose. These research tools, along with clinical trials evaluating potential therapies, will help advance the development of CVMTs and preventive actions against hrHPV-induced neoplasia, which affects thousands of women worldwide.
